# miR-195-5p is critical in REGγ-mediated regulation of wnt/β-catenin pathway in renal cell carcinoma

**DOI:** 10.18632/oncotarget.19256

**Published:** 2017-07-15

**Authors:** Shaojun Chen, Longsheng Wang, Xudong Yao, Hui Chen, Chen Xu, Lu Tong, Abdussaboor Shah, Tingmei Huang, Geng Chen, Jiwei Chen, Tie-Long Liu, Xiao-Tao Li, Jun-Hua Zheng, Lei Li

**Affiliations:** ^1^ Department of Urology, Shanghai Tenth People's Hospital, Tongji University, Shanghai 200072, China; ^2^ Shanghai Key Laboratory of Regulatory Biology, Institute of Biomedical Sciences, School of Life Sciences, East China Normal University, Shanghai 200241, China; ^3^ Department of Orthopedic Oncology, Changzheng Hospital, The Second Military Medical University, Shanghai 200003, China; ^4^ Department of Molecular and Cellular Biology, Dan L. Duncan Cancer Center, Baylor College of Medicine, Houston, Texas 77030, USA

**Keywords:** miR-195-5p, REGγ, wnt/β-catenin, renal cell carcinoma

## Abstract

Renal cell carcinoma (RCC) is the most prevalent malignancy of kidney and accounts for approximately 4% of all cancer diagnoses in adults. Previous studies demonstrated microRNA-195-5p (miR-195-5p) as a tumor suppressor which is deregulated in many human cancers. However, the role of miR-195-5p in RCC is largely unknown. In the present study, we demonstrated that miR-195-5p was downregulated and negatively correlated with advanced clinical stage in RCC. Overexpression of miR-195-5p significantly suppressed RCC cells growth *in vitro* and *in vivo*, induced apoptosis and enhanced chemosensitivity to sorafenib. Conversely, suppression of miR-195-5p exhibited a reverse effect. REGγ, a proteasome activator, was identified as a novel downstream target of miR-195-5p in RCC. Knockdown of REGγ inhibited proliferation, induced apoptosis, increased sorafenib chemosensitivity and suppressed the wnt/β-catenin pathway in RCC cells. Moreover, restoration of REGγ markedly abolished the effects of miR-195-5p in RCC, and the wnt/β-catenin pathway was suppressed by miR-195-5p overexpression while activated by miR-195-5p inhibition in RCC cells. Our findings suggest that miR-195-5p is critical in REGγ-mediated regulation of wnt/β-catenin pathway in RCC development and may serve as a novel target for RCC treatment.

## INTRODUCTION

Renal cell carcinoma (RCC) is the most prevalent malignancy of kidney and accounts for approximately 4% of all cancer diagnoses in adults [1]. At present, surgical resection remains the most effective treatment method for RCC, due to the high resistance of the cancer to conventional chemotherapy and radiotherapy; however, approximately 20-30% of post-surgery treatment patients eventually develop local recurrence or distant metastasis [2, 3]. It is estimated that one third of patients present with metastatic disease when first diagnosed with RCC, and the 5-year survival rate of metastatic RCC is less than 10% [4, 5]. Therefore, the identification of novel therapeutic molecules for RCC may help understanding the pathogenesis of the disease and improving the outcome of the patients.

MicroRNAs (miRNAs) are a class of endogenous, approximately 22 nucleotides in length, single strand noncoding RNAs that regulate gene expression by binding to the 3′untranslated region (UTR) of the corresponding target genes [6, 7]. miRNAs have been shown to be deregulated in various human cancers and may function as oncogenes or tumor suppressors in cancer progression [8]. microRNA-195-5p (miR-195-5p) is a member of the microRNA-15a/b/16/195/497 family that is located on chromosome 17p13.1 [9]. Recently, the aberrant expression of miR-195-5p and its tumor suppressive role were demonstrated in a majority of human cancers namely, breast cancer [10], prostate cancer [11] and hepatocellular carcinoma [12]. However, the function of miR-195-5p in RCC is largely unknown.

REGgamma (REGγ), also known as PSME3, or PA28gamma, was first discovered in the sera of systemic lupus erythematosus patients and identified as the Ki antigen [13]. It is a member of the 11S proteasome activator family that can stimulate the proteolytic activity of the 20S core proteasome, independent of ubiquitination and ATP consumption [14, 15]. REGγ is overexpressed in multiple human cancers, such as colorectal cancer [16], breast cancer [17], thyroid cancer [18] and hepatocellular carcinoma [19], suggesting the potential roles of this protein in cancer development.

In the present study, we found that miR-195-5p was downregulated and negatively correlated with advanced clinical stage in RCC. miR-195-5p suppressed cell growth, induced apoptosis and enhanced chemosensitivity to sorafenib via REGγ-mediated regulation of the Wnt/β-catenin pathway in renal cell carcinoma. Our findings indicated that miR-195-5p may act as a tumor suppressor in RCC and may serve as a novel therapeutic target in RCC treatment.

## RESULTS

### miR-195-5p was downregulated and negatively correlated with advanced clinical stage in RCC

**Figure 1:**
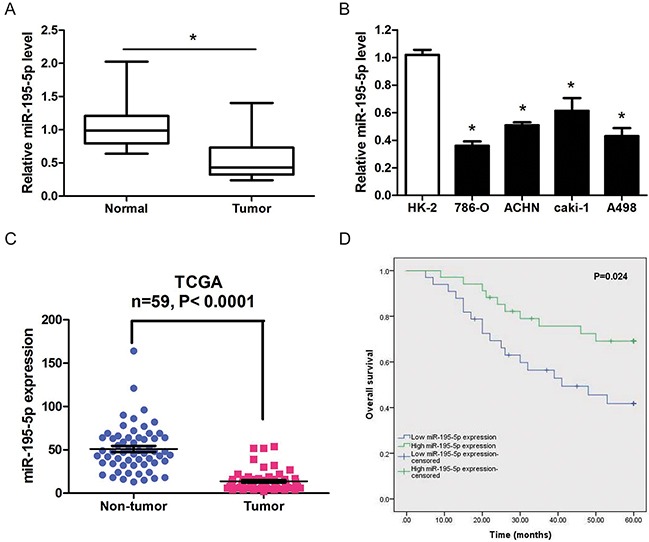
miR-195-5p was downregulated in RCC **(A)** Relative miR-195-5p expression levels in 67 paired RCC tumor tissues and matched normal tissues as determined by qRT-PCR. **(B)** Relative miR-195-5p levels in 4 different RCC cell lines and the immortalized primary human proximal tubular cell line HK-2 as determined by qRT-PCR. U6 was used as internal control. **(C)** Downregulation of miR-195-5p in RCC tissues confirmed by TCGA database (n=59, P<0.0001). **(D)** Kaplan-Meier survival rates for RCC patients with low and high miR-195-5p expression. The data are presented as mean ± SD of 3 independent experiments. *P < 0.05

The expression of miR-195-5p was determined in 67 paired ccRCC tumor tissues and matched normal tissues in order to elucidate the potential role of miR-195-5p in the development of RCC. The results showed that miR-195-5p was significantly downregulated in RCC tumor tissues compared with adjacent normal tissues (Figure 1A). And, we found that the miR-195-5p expression levels in 4 RCC cell lines (786-O, ACHN, caki-1 and A498) were markedly decreased compared to the immortalized primary human proximal tubular cell line HK-2 (Figure 1B). In addition, TCGA database also indicated a significant lower miR-195-5p expression in RCC compared with non-tumor tissues (Figure 1C). Next we investigated the realtionship between miR-195-5p expression level and clinicopathologic factors in the 67 ccRCC patients. We found that miR-195-5p expression was significantly correlated with clinical stage, histological grade, tumor stage and lymph node metastasis (P<0.05), but was not significant associated with patients’ gender, age and tumor size (Table 1). Moreover, Kaplan–Meier analysis revealed that ccRCC patients with low miR-195-5p expression levels had a significantly shorter overall survival time than those with high miR-195-5p levels (Figure 1D). These results indicated that miR-195-5p may play a tumor suppressive role in renal cell carcinoma.

**Table 1:** Relationship between miR-195-5p expression level and clinicopathologic factors

Parameters	Group	Total	miR-195-5p expression		*P value*
			High	Low	
Gender	Male	38	16	22	0.180
	Female	29	17	12	
Age (years)	<65	32	18	14	0.273
	≥65	35	15	20	
Tumor size (cm)	<4 cm	25	11	14	0.507
	≥4 cm	42	22	20	
Clinical stage	I–II	39	25	14	0.004
	III-IV	28	8	20	
Histological grade	G1-G2	47	28	19	0.010
	G3-G4	20	5	15	
Tumor stage	T1-T2	42	25	17	0.029
	T3-T4	25	8	17	
Lymph nodes metastasis	Absence	49	29	20	0.007
	Presence	18	4	14	

### miR-195-5p suppressed RCC cell growth *in vitro* and xenograft tumor growth *in vivo*

**Figure 2:**
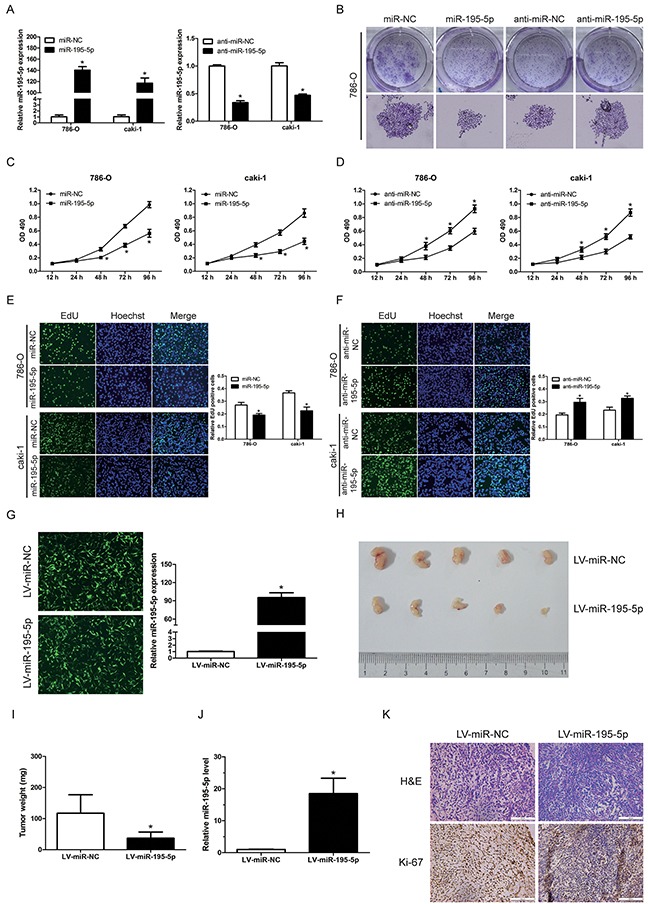
miR-195-5p suppressed RCC cell growth *in vitro* and xenograft tumor growth *in vivo* **(A)** 786-O and caki-1 cells were transfected with miR-195-5p mimics (miR-195-5p) or inhibitors (anti-miR-195-5p), and their negative controls (miR-NC or anti-miR-NC). The relative miR-195-5p levels were determined by qRT-PCR following 48 h of culture. **(B)** Colony formation of 786-O cells following transfection of miR-195-5p mimics or inhibitor. **(C and D)** The effects of miR-195-5p mimics (C) or inhibitors (D) on 786-O and caki-1 cells proliferation as demonstrated by the MTT assay. **(E and F)** The effects of miR-195-5p mimics (E) or inhibitors (F) on 786-O and caki-1 cells proliferation as demonstrated by the EdU proliferation assay. The cells with green fluorescence are in the S phase of mitosis, and the cells with blue fluorescence represent the entire cell population. **(G)** 786-O cells were infected with miR-195-5p overexpressing lentivirus (LV-miR-195-5p) or the negative control lentivirus (LV-miR-NC). The images were captured using a fluorescence microscope at a magnification of 100×, and the relative miR-195-5p expression was evaluated by qRT-PCR. **(H and I)** Following infection with LV-miR-195-5p or LV-miR-NC, 786-O cells were implanted subcutaneously in 6-week-old nude mice. The tumor growth was evaluated following 3 weeks of the tumor implantation. Representative images (H) and weight (I) of the excised tumors derived from nude mice are shown. **(J)** The relative miR-195-5p levels in xenograft tumors as determined by qRT-PCR. **(K)** Representative images of H&E staining and Ki-67 immunohistochemical detection of the excised tumors derived from nude mice. Scale bar = 100 μ m. Data are shown as mean ± SD of 3 independent experiments. *P < 0.05

In order to determine the function of miR-195-5p in RCC, miR-195-5p mimics (miR-195-5p) or inhibitors (anti-miR-195-5p) were transfected into 2 renal carcinoma cell lines (786-O and caki-1) respectively, combined with their negative controls (miR-NC or anti-miR-NC) (Figure 2A). Subsequently, the results of colony formation assay indicated that the growth of 786-O cells was significantly suppressed following transfection with miR-195-5p mimics. Conversely, the cells transfected with miR-195-5p inhibitors showed significantly higher colony formation rates compared with the NC inhibitor (Figure 2B). Moreover, MTT and EdU proliferation assays indicated that overexpression of miR-195-5p significantly inhibited proliferation in RCC cells (Figure 2C, 2E), while suppression of miR-195-5p markedly enhanced RCC cells proliferation (Figure 2D, 2F).

To further define the suppressive role of miR-195-5p in renal cell carcinoma. 786-O cells were infected with a miR-195-5p lentiviral expression vector (LV-miR-195-5p) to overexpress miR-195-5p or the negative control lentivirus (LV-miR-NC). The infection efficiency was examined by fluorescent microscopy and miR-195-5p expression was evaluated by qRT-PCR (Figure 2G). The cells were subsequently collected and implanted subcutaneously in 6-week-old nude mice. The xenograft tumor growth was estimated following 3 weeks of tumor implantation. A significant decrease in the size and weight of tumors was observed in the miR-195-5p overexpressed group compared with that in the control group (Figure 2H, 2I). The relative miR-195-5p levels in xenograft tumors were confirmed to be upregulated in LV-miR-195-5p groups (Figure 2J). The number of Ki-67-positive cells in tumors from miR-195-5p overexpression group was lower than that in the control group as shown by immunohistochemistry (Figure 2K), indicating a tumor suppressor potential.

### miR-195-5p induced apoptosis and cell cycle arrest in RCC cells

**Figure 3:**
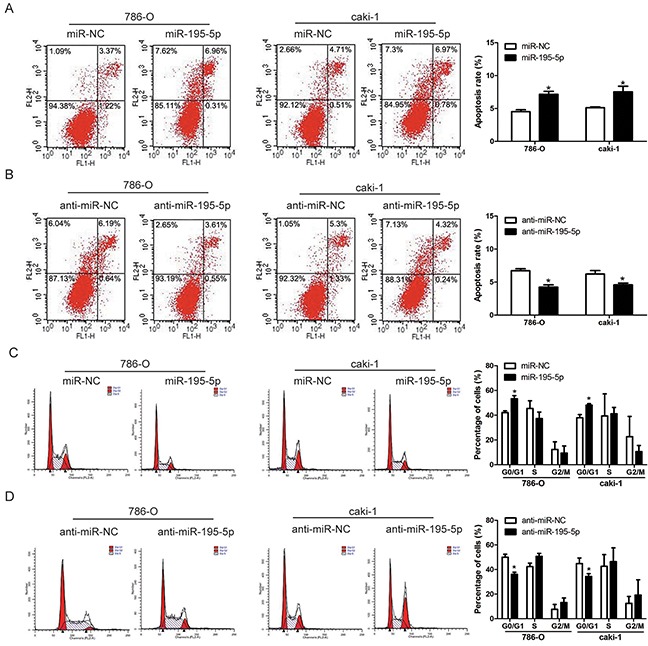
miR-195-5p induced apoptosis and cell cycle arrest in RCC **(A and B)** Apoptosis of 786-O and caki-1 cells as measured by flow cytometry following miR-195-5p overexpression (A) or suppression (B). **(C and D)** The effects of miR-195-5p mimics (C) or inhibitors (D) on the cell cycle distribution of 786-O and caki-1 cells as determined by flow cytometry. The data are presented as mean ± SD of 3 independent experiments. *P < 0.05

The effects of miR-195-5p on RCC cells apoptosis and cell cycle distribution were determined by flow cytometry. Our results revealed an obvious increase of cell apoptosis in 786-O and caki-1 cells after overexpression of miR-195-5p, whereas suppression of miR-195-5p significantly inhibited RCC cells apoptosis (Figure 3A, 3B). In addition, cell cycle analysis indicated that the percentage of cells in G0/G1 phase was markedly higher in the miR-195-5p mimics group and lower in the miR-195-5p inhibitors group compared with the control groups (Figure 3C, 3D). These results suggested that miR-195-5p may suppress cell growth by inducing apoptosis and cell cycle arrest in RCC cells.

### miR-195-5p enhanced RCC cells chemosensitivity to sorafenib

**Figure 4:**
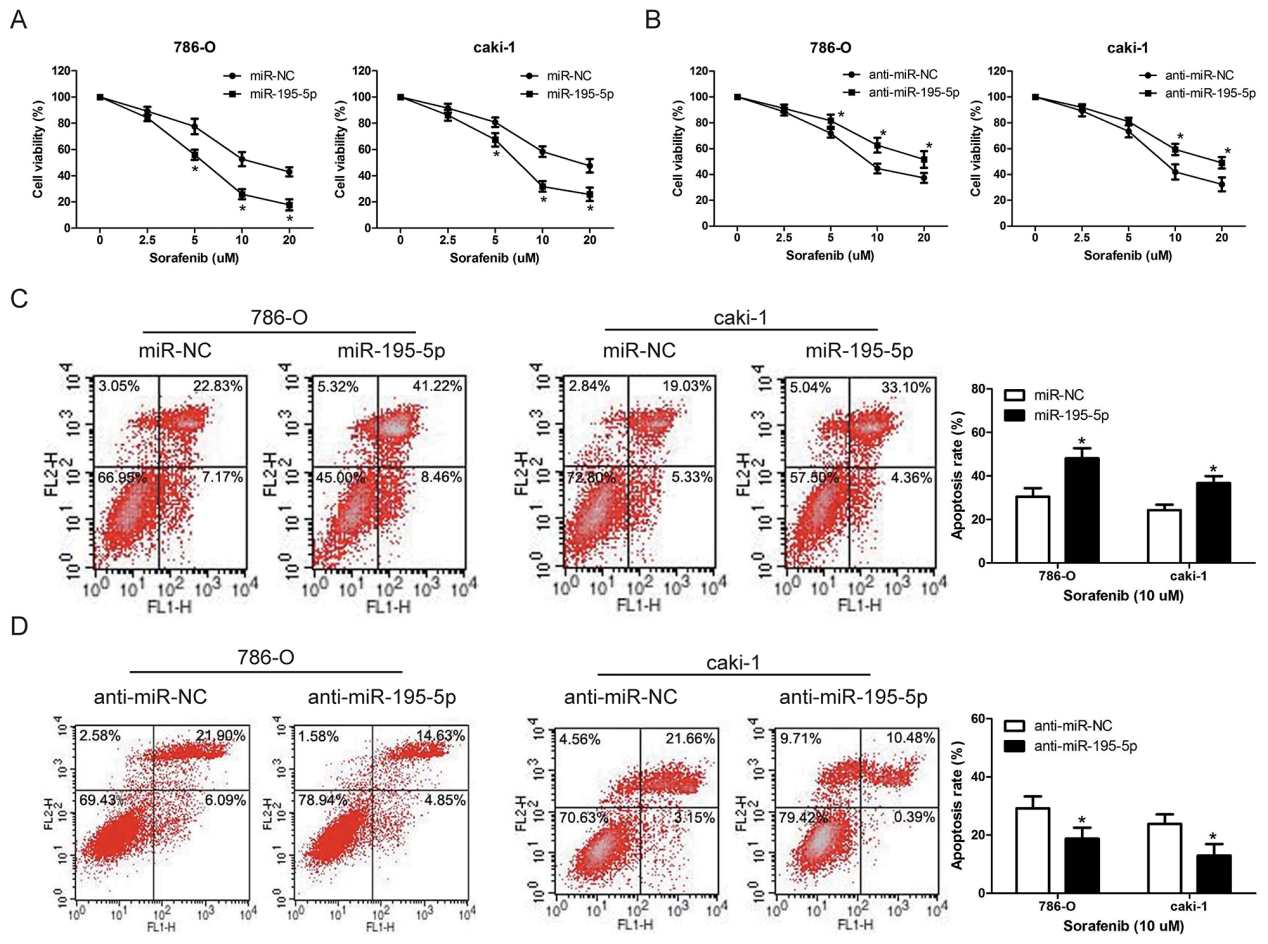
miR-195-5p enhanced RCC cells chemosensitivity to sorafenib **(A and B)** 786-O and caki-1 cells were transfected with miR-195-5p mimics (A) or inhibitors (B), and then cultured with sorafenib at various concentrations of 0, 2. 5, 5, 10, 20 μM for 24 h. Subsequently, cell viability was determined by the MTT assay. **(C and D)** 786-O and caki-1 cells were transfected with miR-195-5p mimics (C) or inhibitors (D), treated with sorafenib at the concentration of 10 μM for 24 h, and apoptosis was measured by flow cytometry. The data are presented as mean ± SD of 3 independent experiments. *P < 0.05

The standard first-line treatment for advanced RCC is the targeted therapy and sorafenib is widely used for targeted therapy in RCC patients. As miR-195-5p exhibited a tumor suppressive role in RCC progression, we further investigated the effect of miR-195-5p on RCC cells chemosensitivity to sorafenib. Following overexpression or suppression of miR-195-5p, the RCC cell lines 786-O and caki-1 were treated with various concentrations of sorafenib (0 ∼ 20 μM) for 24 h and the cell viability was evaluated by MTT assay. The results indicated that chemosensitivity to sorafenib was significantly increased by miR-195-5p overexpression, while decreased by miR-195-5p suppression in both 786-O and caki-1 cells (Figure 4A, 4B). Similarly, the measurement of apoptosis induction that occurred after 24 h of sorafenib treatment (10 μM) also showed that miR-195-5p enhanced the chemosensitivity of RCC cells to sorafenib (Figure 4C, 4D)

### REGγ is a direct downstream target of miR-195-5p in RCC

**Figure 5:**
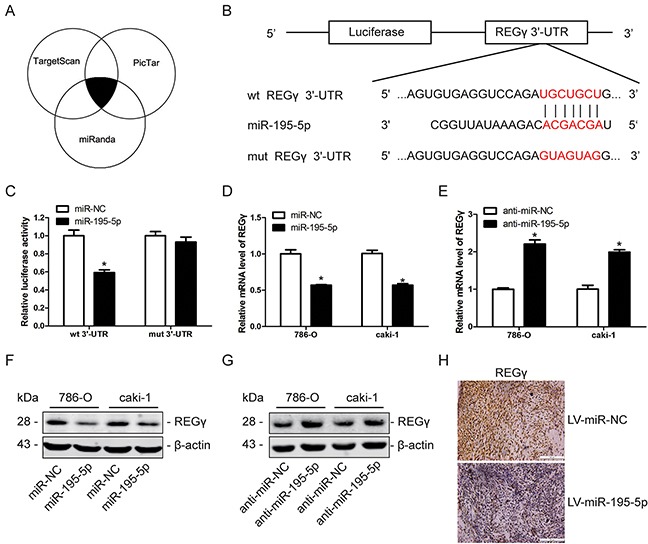
REGγ is a direct downstream target of miR-195-5p in RCC **(A)** REGγ was predicted as a direct target of miR-195-5p by bioinformatic analysis using the TargetScan, PicTar and miRanda databases. The overlap represents the same prediction derived by 3 databases. **(B)** The sequences of miR-195-5p binding sites in the 3′-UTR of wild-type (wt) REGγ mRNA and of a mutant (mut) 3′-UTR of REGγ. **(C)** The relative luciferase activity of the wt REGγ 3′-UTR and mut REGγ 3′-UTR reporters in 786-O cells in the presence of miR-195-5p mimics (miR-195-5p) or the negative control (miR-NC). **(D and E)** The relative REGγ mRNA levels in 786-O and caki-1 cells as determined by qRT-PCR following transfection with miR-195-5p mimics (D) or inhibitors (E). 18S was used as an internal control. **(F and G)** Western blot measurement of REGγ protein expression in 786-O and caki-1 cells following transfection with miR-195-5p mimics (F) or inhibitors (G). **(H)** The expression of REGγ was evaluated by immunohistochemistry in tumor tissues derived from a xenograft nude mice model. Scale bar= 100 μm. The data are presented as mean ± SD of 3 independent experiments. *P < 0.05

A bioinformatics approach was used to understand the mechanism of miR-195-5p in the suppression of RCC progression. A total of 3 bioinformatic databases including TargetScan, PicTar and miRanda were used in combination to predict the putative targets of miR-195-5p (Figure 5A). REGγ, which was reported as an oncogene in many human cancers, was selected as a potential target of miR-195-5p among the screened candidate genes (Figure 5B). Dual-luciferase reporter assay, qRT-PCR and western blot analysis were carried out to confirm the relationship between REGγ and miR-195-5p. Dual-luciferase reporter assay showed that the luciferase activity in 786-O cells was significantly reduced when co-transfected with miR-195-5p mimics and the wild type (wt) but not the mutant (mut) 3′-UTR of REGγ (Figure 5C). This finding indicated that miR-195-5p could directly bind to the 3′-UTR of REGγ in RCC. In addition, the qRT-PCR results indicated that the mRNA level of REGγ is down-regulated after transfection with miR-195-5p mimics while up-regulated following transfection with miR-195-5p inhibitors in RCC cells (Figure 5D, 5E). In addition, western blot analysis indicated that the REGγ protein level was downregulated following overexpression of miR-195-5p while upregulated following suppression of miR-195-5p (Figure 5F, 5G). Furthermore, the immunohistochemical analysis of the xenograft tumor tissues generated from LV-miR-195-5p and LV-miR-NC 786-O cells revealed a marked reduction of REGγ expression when miR-195-5p was overexpressed (Figure 5H). These findings collectively suggest that REGγ is a direct downstream target of miR-195-5p in RCC.

### Knockdown of REGγ inhibited proliferation and increased chemosensitivity to sorafenib by suppressing the wnt/β-catenin pathway in RCC cells

**Figure 6:**
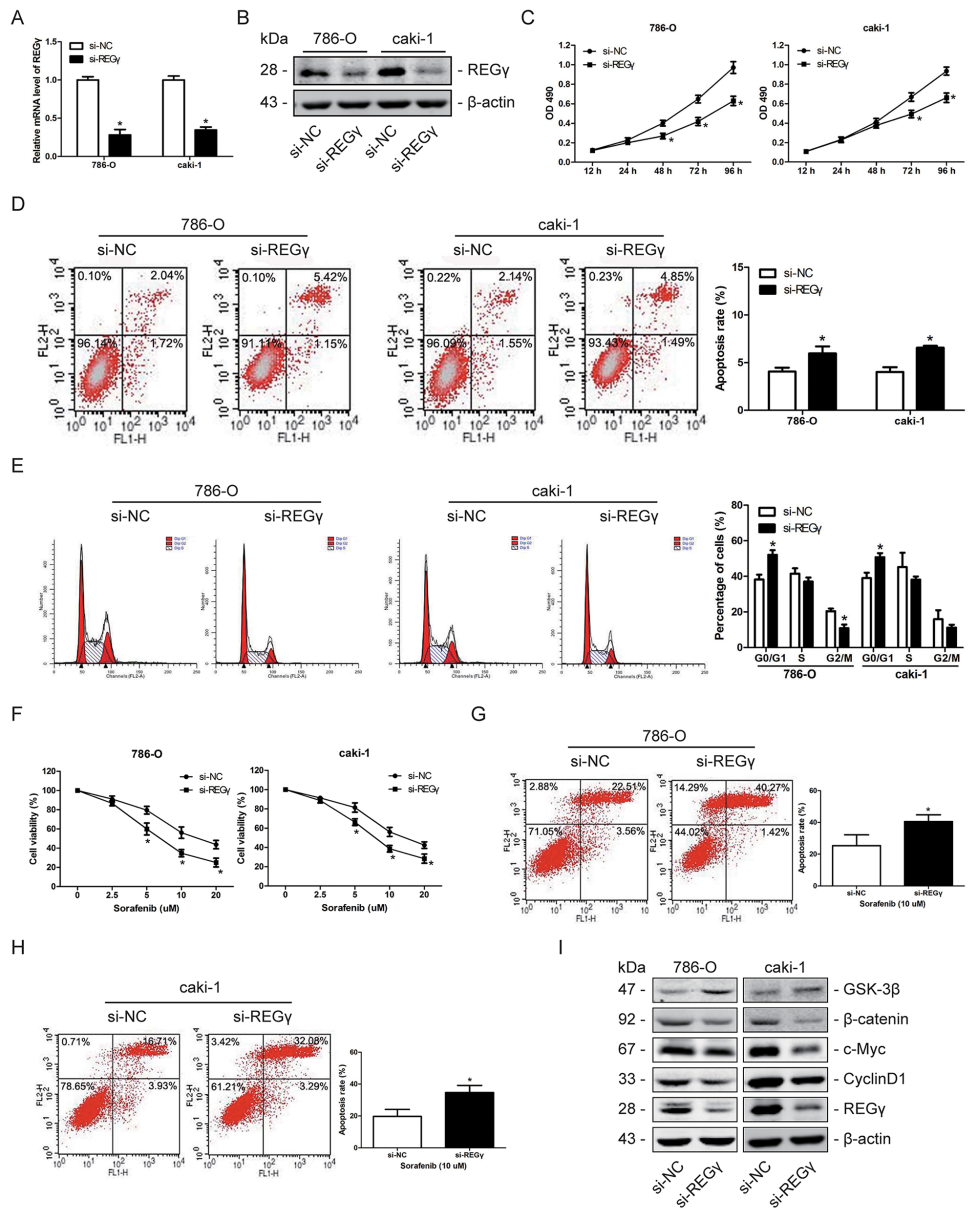
Knockdown of REGγ inhibited proliferation and increased chemosensitivity to sorafenib by suppressing the wnt/β-catenin pathway in RCC cells **(A and B)** 786-O and caki-1 cells were transfected with si-REGγ or si-NC. The relative mRNA levels (A) and the protein expression (B) of REGγ were determined by qRT-PCR and Western blot analysis, respectively. **(C)** The proliferation of 786-O and caki-1 cells was analyzed by the MTT assay following REGγ knockdown. **(D)** The apoptosis rate of 786-O and caki-1 cells was determined by flow cytometry following REGγ knockdown. **(E)** The cell cycle distribution of 786-O and caki-1 cells was measured by flow cytometry following REGγ knockdown. **(F)** 786-O and caki-1 cells were transfected with si- REGγ or si-NC, and further cultured with sorafenib at various concentrations of 0, 2.5, 5, 10, 20 μM. Following 24 h of sorafenib treatment, cell viability was determined by the MTT assay. **(G and H)** 786-O and caki-1 cells were transfected with si-REGγ or si-NC, further treated with sorafenib at the concentration of 10 μM for 24 h, and apoptosis was measured by flow cytometry. **(I)** Western blot measurement of GSK-3β, β-catenin, c-Myc and Cyclin D1 protein expression levels following knockdown of REGγ in 2 RCC cells. β-actin was used as an internal control. The data are presented as mean ± SD of 3 independent experiments. *P < 0.05

REGγ expression was knocked down by siRNA and the efficiency was confirmed by qRT-PCR and western blot analysis (Figure 6A, 6B). The results of the MTT assay demonstrated that knockdown of REGγ significantly inhibited cell proliferation of both 786-O and caki-1 cell lines (Figure 6C). Flow cytometry results indicated that knockdown of REGγ obviously induced apoptosis (Figure 6D) and cell cycle arrest (Figure 6E) in RCC cells. Moreover, we estimated chemosensitivity of 786-O and caki cells to sorafenib following REGγ knockdown as described. After sorafenib treatment, cell viability and apoptosis were determined by the MTT assay (Figure 6F) and flow cytometry (Figure 6G, 6H), respectively. The results indicated that REGγ knockdown increased RCC cells chemosensitivity to sorafenib.

We have reported before that REGγ is involved in skin carcinogenesis via direct degradation of GSK-3β and modulation of the Wnt/β-catenin pathway in our previous study [20]. And the significant role of the Wnt/β-catenin signaling in RCC initiation and development has also been well addressed by previous studies [21]. Moreover, previous studies have showed that Wnt/β-catenin pathway is involved with sorafenib sensitivity [22, 23]. We presumed that REGγ may regulate Wnt/β-catenin pathway to exert its effects in RCC as well and conducted western blot analysis to verify that. We observed an increased GSK-3β level, while β-catenin, c-Myc and CyclinD1 were decreased in RCC cells following knockdown of REGγ (Figure 6I). These results suggested that knockdown of REGγ inhibited cell proliferation and increased chemosensitivity to sorafenib by suppressing the wnt/β-catenin pathway in RCC.

### Restoration of REGγ markedly abolished the effects of miR-195-5p in RCC

**Figure 7:**
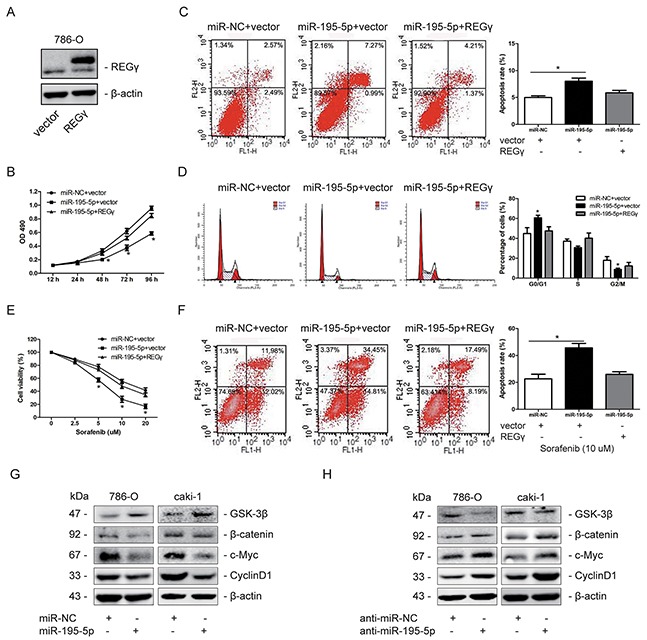
Restoration of REGγ markedly abolished the effects of miR-195-5p in RCC **(A)** Restoration of REGγ in 786-O cells determined by western blots. **(B)** 786-O cells were transfected with miR-NC or miR-195-5p mimics and pcDNA5-REGγ plasmid or empty vector, and cell proliferation was analyzed by MTT assay. **(C and D)** Flow cytometry was performed on transfected 786-O cells to measure the apoptosis rate and cell cycle distribution, respectively. **(E)** Following transfection, 786-O cells were cultured with sorafenib at various concentrations of 0, 2.5, 5, 10, 20 μM for 24 h. And cell viability was determined by the MTT assay. **(F)** The transfected 786-O cells were treated with sorafenib at the concentration of 10 μM for 24 h, and apoptosis was measured by flow cytometry. **(G and H)** Western blot measurement of GSK-3β, β-catenin, c-Myc and Cyclin D1 protein expression levels following miR-195-5p overexpression (G) or suppression (H) in 786-O and caki-1 cells. β-actin was used as an internal control. The data are presented as mean ± SD of 3 independent experiments. *P < 0.05

To further confirm whether the roles of miR-195-5p in RCC was mediated by REGγ, we transfected pcDNA5-REGγ plasmid to restoring REGγ expression when miR-195-5p was overexpressed in 786-O cells (Figure 7A). Data showed that restoration of REGγ significantly abolished the effects of miR-195-5p on RCC cells proliferation, apoptosis and cell cycle distribution (Figure 7B, 7C and 7D). Consistently, the increase of chemosensitivity to sorafenib by miR-195-5p was also abolished by REGγ restoration in 786-O cells (Figure 7E, 7F). In addition, we determined the effect of miR-195-5p on Wnt/β-catenin pathway. Our results revealed that overexpression of miR-195-5p significantly suppressed Wnt/β-catenin pathway, while suppression of miR-195-5p exhibited an opposite results in RCC cells (Figure 7G, 7H). These results collectively suggested that miR-195-5p may exert its roles via REGγ-mediated regulation of Wnt/β-catenin pathway in RCC.

## DISCUSSION

An increasing number of studies have shown that dysregulation of miRNAs is a common event in human cancers including renal cell carcinoma. miR-195-5p was reported as a tumor suppressor in various human cancers. In our study, we found that miR-195-5p was significantly downregulated and negatively correlated with advanced clinical stage in RCC. That was consist with a previous study which reported that miR-195-5p expression is deceased in RCC by using TaqMan Low Density Arrays [24]. In addition, patients with low miR-195-5p expression level had a significantly shorter overall survival time than those with high miR-195-5p level, indicating the potential value of miR-195-5p as a prognosis predictor in RCC patients. The overexpression of miR-195-5p significantly inhibited cellular proliferation, in accordance with induction of apoptosis and cell cycle arrest in 786-O and caki-1 RCC cells. In addition, inhibition of miR-195-5p had a converse effect on RCC cellular proliferation, apoptosis and cell cycle distribution. Furthermore, overexpression of miR-195-5p significantly suppressed RCC tumorigenesis in a xenograft nude mice model. Our results indicated that miR-195-5p acted as a tumor suppressor in RCC development.

The resistance to chemotherapy drugs remains the major impediment towards successful cancer treatment. Emerging evidence suggests that miRNAs can modulate chemosensitivity via regulation of multiple target genes. For example, miRNA-185-5p was shown to modulate chemosensitivity of human non-small cell lung cancer to cisplatin via targeting ABCC1 [25]. While miRNA-155 can regulate cell survival, growth and chemosensitivity by targeting FOXO3a in breast cancer [26]. In addition, miR-146a was reported to enhance chemosensitivity in epithelial ovarian cancer via reduction of SOD2 expression [27]. In accordance with the aforementioned findings, miR-195-5p was also demonstrated to modulate chemosensitivity in various human cancers. It was reported that miRNA-195-5p sensitizes human hepatocellular carcinoma cells to 5-FU by targeting BCL-w [28]. In breast cancer, miR-195-5p can increase the sensitivity of the cells to adriamycin treatment via Raf-1 inhibition [29]. Qu et al. also demonstrated that miRNA-195-5p chemosensitizes colon cancer cells to doxorubicin by targeting BCL2L2 [30]. As miR-195-5p acted as a tumor suppressor in RCC development, the effect of miR-195-5p in the modulation of chemosensitivity to sorafenib in RCC cells was investigated. In our study, miR-195-5p was shown to enhance the chemosensitivity of RCC cells to sorafenib by measuring cell viability and apoptosis following sorafenib treatment.

To explore the mechanism by which miR-195-5p exerts its effect in RCC, the potential target genes of miR-195-5p were predicted by 3 bioinformatic databases and REGγ was selected as a candidate gene. By using Dual-luciferase reporter assay, qRT-PCR, western blot analysis and immunohistochemical staining of the xenograft tumor tissues, we demonstrated that miR-195-5p could directly bind to the 3′-UTR of REGγ and suppress its expression in RCC. In addition, knockdown of REGγ significantly inhibited proliferation, induced apoptosis and increased sorafenib chemosensitivity in RCC cells, and restoration of REGγ markedly abolished the effects of miR-195-5p in RCC. These results suggest that miR-195-5p may exert its function by targeting REGγ in RCC.

In skin carcinogenesis, we have showed that REGγ could modulate the Wnt/β-catenin pathway [20], which is also crucial in RCC development. The regulation of Wnt/β-catenin pathway by REGγ in RCC was estimated by western blot analysis. We observed that knockdown of REGγ markedly suppressed the wnt/β-catenin pathway in RCC cells. Given that REGγ could regulate the Wnt/β-catenin pathway and that REGγ was a direct target of miR-195-5p in RCC, we presumed that miR-195-5p may have effect on wnt/β-catenin pathway in RCC. Results of western blot showed that the wnt/β-catenin pathway was suppressed by miR-195-5p overexpression while activated by miR-195-5p inhibition in RCC cells. To the best of our knowledge, miRNA-195-5p was reported to suppress colorectal cancer cell proliferation via regulation of FGF2 and the Wnt/β-catenin pathway [31], indicating that the latter pathway may be involved in the tumor suppressive function of miR-195-5p. Our results suggest that miR-195-5p is critical in REGγ-mediated regulation of wnt/β-catenin pathway in renal cell carcinoma.

**Figure 8:**
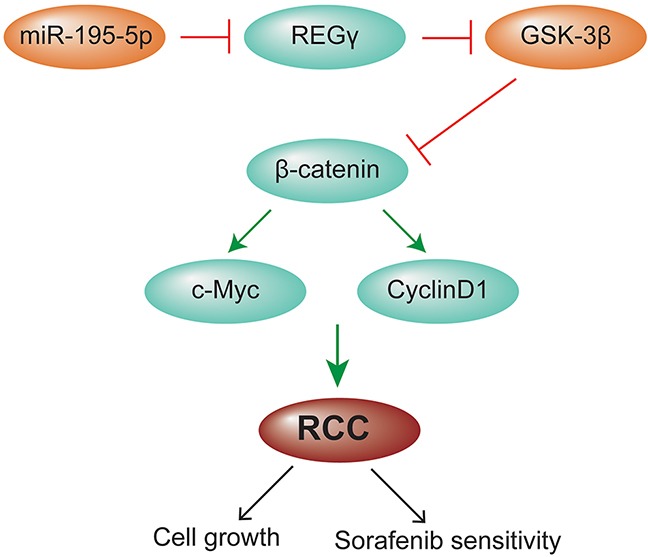
A schematic model for the effect of miR-195-5p in RCC miR-195-5p suppressed cell growth and enhanced chemosensitivity to sorafenib via REGγ mediated regulation of the Wnt/β-catenin pathway in renal cell carcinoma.

In summary, our study demonstrated that miR-195-5p was downregulated in RCC tissue samples compared with matched normal tissues. miR-195-5p suppressed cell growth, induced apoptosis and enhanced chemosensitivity to sorafenib via REGγ-mediated regulation of the Wnt/β-catenin pathway in renal cell carcinoma, which was summarized by the schematic model (Figure 8). Our findings suggested that miR-195-5p acted as a tumor suppressor in RCC and may serve as a new target for RCC therapy.

## MATERIALS AND METHODS

### Reagents

Antibodies were purchased from Invitrogen (REGγ), Proteintech (GSK-3β), Abcam (c-Myc, CyclinD1, β-actin, Ki-67) and Cell Signaling Technology (β-catenin). Fluorescent-labelled secondary antibodies were purchased from Jackson Immuno Research. Sorafenib was purchased from Selleckchem (Houston, TX, USA).

### Tissue samples

All tumor tissues and adjacent normal tissues were obtained from 67 patients with primary clear cell RCC (ccRCC) who underwent radical nephrectomy at the Department of Urology, Shanghai Tenth People's Hospital, Tongji University from 2007 to 2010. None of the patients received preoperative treatment. All samples were immediately snap-frozen in liquid nitrogen following surgery, and stored in liquid nitrogen until further use. Our work has been carried out in accordance with The Code of Ethics of the World Medical Association (Declaration of Helsinki). The study was approved by the Ethics Committees of Shanghai Tenth People's Hospital and informed consent was obtained from each patient prior to his/her participation in the study.

### Cell culture

Human RCC cell lines (786-O, ACHN, caki-1 and A498) were obtained from the Institute of Cell Research of the Chinese Academy of Sciences (Shanghai, China). The immortalized primary human proximal tubular cell line (HK-2) was obtained from the American Type Culture Collection (ATCC, Rockville, USA). The cell lines 786-O and caki-1 were cultured in RPMI-1640 medium (Gibco, USA), ACHN and A498 cells were cultured in Dulbecco's modified Eagle's medium (DMEM, Gibco) and HK-2 was cultured in F-12K medium (Gibco). All media were supplemented with 10% fetal bovine serum (Gibco), 50 U/ml of penicillin and 50 μg/ml of streptomycin (Invitrogen, USA). Cells were all incubated in a humidified incubator containing 5% CO_2_ at 37°C.

### RNA isolation and qRT-PCR

Total RNA was extracted from tissues or cells using TRIzol reagent (Invitrogen) and reversely transcribed into cDNA with M-MLV reverse transcriptase (Invitrogen) according to the manufacturer's protocol. Quantitative real time-PCR (qRT-PCR) was conducted on Applied Biosystems 7900HT (Applied Biosystems) using SYBR green (Invitrogen). U6 and 18s were used as internal controls for miR-195-5p and REGγ, respectively. The primer sequences (Biosune Biotech, Shanghai, China) were as follows: 5′-GTCGTATCCAGTGCGTGTCGTGGAGTCGGCAATTGCACTGGA TACGACGCCAAT-3′ (stem-loop primer), 5′-GGGGTA GCAGCACAGAAAT-3′ (sense) and 5′-TCCAGTGC GTGTCGTGGA-3′ (antisense) for miR-195-5p; 5′-GTCCTATCCAGTGCAGGGTCCGAGGTGCACTGGATACGACAAAATATGGAAC-3′ (stem-loop primer) 5′-TG CGGGTGCTCGCTTCGCAGC-3′ (sense) and 5′-CCA GTGCAGGGTCCGAGGT-3′ (antisense) for U6; 5′-AAGGTTGATTCTTTCAGGGAGC-3′ (sense) and 5′-AGTGGATCTGAGTTAGGTCATGG-3′ (antisense) for REGγ; 5′-GGACACGGACAGGATTGACA-3′ (sense) and 5′-GACATCTAAGGGCATCACCAG-3′ (antisense) for 18S. The 2^−ΔΔCt^ method was used to determine the relative quantitation of miRNA and mRNA expression. All assays were conducted in triplicate.

### Transient transfection

miR-195-5p mimics (miR-195-5p), inhibitors (anti-miR-195-5p), siRNA targeting human REGγ (si-REGγ) and non-specific negative control oligos (miR-NC, anti-miR-NC and si-NC) were all purchased from GenePharma (Shanghai, China). pcDNA5-REGγ plasmid and the empty vector were constructed before. Cells were transfected using Lipofectamine 2000 (Invitrogen) following the manufacturer's instructions. The sequences are 5′-UAGCAGCACAGAAAUAUUGGC-3′ for miR-195-5p mimics, 5′-UUCUCCGAACGUGUCACGUTT-3′ for miR-NC, 5′ –GCCAAUAUUUCUGUGCUGCUA-3′ for miR-195-5p inhibitors and 5′-CAGUACUUUUGU GUAGUACAA-3′ for inhibitor NC. The sequences of si-REGγ were 5′-CAGAAGACUUGGUGGCAAATT-3′ (sense) and 5′-UUUGCCACCAAGUCUUCUGTT-3′ (antisense). The sequences of si- NC were 5′-UUCU CCGAACGUGUCACGUTT-3′ (sense) and 5′-ACGU GACACGUUCGGAGAATT-3′ (antisense). Total RNA or protein was extracted 48 h following transfection.

### MTT assay and colony formation assay

Cell proliferation was measured by the 3-(4, 5-dimethyl-2-thiazolyl)-2, 5-diphenyltetrazolium bromide (MTT) assay according to the manufacturer's guideline. In brief, cells were seeded into 96-well plates and incubation at 37°C for different time periods (12 h, 24 h, 48 h, 72 h, 96 h). Then, 100 μl of full medium containing 0.5 mg/mL MTT (Sigma-Aldrich, St. Louis, Mo, USA) were added to each well and incubated for a further 4 h. The supernatant was then discarded and 150 μl DMSO (Sigma-Aldrich) was added to resolve the crystals. The optical density (OD) values were measured at 490 nm (SpectraMax 190; Molecular Devices Sunnyvale, CA, USA).

For colony formation assays, cells were transfected with a miR-195-5p mimic or inhibitors for 48 h and then plated in 6-well plates at a density of 1×10^3^/well for 10 days. The plates were washed twice with cold PBS, fixed with methanol and stained with 0.1% crystal violet (0.1% in 20% methanol). The images of stained tumor cell colonies were recorded by a digital camera. Each experiment was conducted in triplicate.

### EdU proliferation assay

Cell proliferation was measured by the incorporation of 5-ethynyl-2′-deoxyuridine (EdU) during DNA synthesis using the EdU Cell Proliferation Assay Kit (Ribobio, Guangzhou, China). The assay was carried out as determined by the manufacture's instruction. Briefly, the transfected cells were seeded in 96-well plates and incubated with 50 μM EdU for 2 h prior to the fixation process. The cell nuclei were stained with Hoechst. Finally, the EdU positive cells were captured and quantified by fluorescence microscopy. Three independent experiments were conducted for each sample.

### Flow cytometry

Cell apoptosis was determined using Annexin V-FITC apoptosis detection kit (BD Biosciences, Erembodegem, Belgium) in accordance with the manufacturer's instructions. Cells were collected, washed twice with cold PBS and resuspended in Annexin V-binding buffer. Subsequently, they were incubated with 5 μl Annexin V-FITC and 5 μl propidium iodide (PI) in the dark at room temperature for 15 min. The apoptosis rate was analyzed by flow cytometry using BD FACS Calibur (Beckman Coulter, CA, USA). Whereas as regards the cell cycle distribution analysis, the cells were collected and resuspended in PBS containing PI and 50 μg/ml RNase A (Sigma-Aldrich) in the dark at 37°C for 30 min. Flow cytometry was conducted to analyze cell cycle distribution. Each experiment was independently repeated 3 times.

### Xenograft assays in nude mice

The hsa-miR-195-5p sequence was cloned into the lentiviral vector PHY-502 carrying a green fluorescent protein (GFP) sequence by Hanyin Co. (Shanghai, China). The recombinant has-miR-195-5p expression lentivirus (LV-miR-195-5p) and the negative control lentivirus (LV- miR-NC; Hanyin Co. Shanghai, China) were prepared and titered to 10^9^ TU/ml (transfection unit). 786-O cells were infected with LV-miR-195-5p or LV-NC and then selected by puromycin (Sigma-Aldrich) in order to generate the stably transfected miR-195-5p overexpressing cell lines. Subsequently, the cells were implanted in the dorsal flanking sites of male BALB/c nude mice (6 weeks) at a density of 2×10^6^ cells in 100 μl of PBS. Following 3 weeks of tumor implantation, mice bearing tumors were sacrificed for the assessment of tumor size, weight and immunohistological examination. Athymic nude mice were provided by Shanghai SLAC Laboratory Animal Co., Ltd (Shanghai, China). The animal care and animal experiments were carried out in accordance with the NIH Guide for the Care and Use of Laboratory Animals.

### Dual-Luciferase reporter assay

The 3′-UTR sequences of human REGγ mRNA, containing the putative miR-195-5p binding site, were amplified by PCR and cloned into the XhoI /BglII site of the pGL3 vector (Promega, Madison, WI, USA) in order to construct the wild-type REGγ 3′-UTR plasmid (wt 3′-UTR). The mutant REGγ 3′-UTR plasmid (mut 3′-UTR) was generated using the Site-Directed Mutagenesis Kit (SBS Genetech, Beijing, China). The cultured 786-O cells were co-transfected with wt or mut REGγ 3′-UTR plasmid, miR-195-5p mimics (miR-195-5p), negative control miR (miR-NC) and a control Renilla luciferase pRL-TK vector (Promega) using Lipofectamine 2000 reagent (Invitrogen). Following 48 h of incubation, the cells were harvested and lysed. The luciferase activity was analyzed using the Dual-Luciferase Reporter Assay System (Promega) according to the manufacturer's protocol. The firefly luciferase fluorescence was normalized to Renilla luminescence from the same vector. Experiments were independently repeated 3 times.

### Western blot

Equal amounts of total protein extracted from cells were loaded on a polyacrylamide gel (10%) and subjected to electrophoresis. The separated proteins were transferred to pure nitrocellulose (NC) membranes and blocked with 5% fat free dry milk in PBS for 1 h at room temperature. The membrane was subsequently incubated with primary antibody at 4°C overnight. After washed three times with PBS-T, membranes were incubated with a fluorescent-labelled secondary antibody (1:5,000 dilutions) for 1 h at 4°C. The specific signals that corresponded to the protein expression were visualized by a LI-COR Odyssey Infrared Imaging System. A total of 3 independent experiments were carried out.

### Immunohistochemistry

Tumors excised from nude mice were fixed in 4% paraformaldehyde, dehydrated through a graded series of ethanol solution and embedded in paraffin. The sections were cut at 4 mm and stained with hematoxylin and eosin (H&E). The sections were incubated with primary antibody versus REGγor Ki-67 overnight at 4°C for immunostaining experiments. Subsequently, the sections were incubated with biotinylated goat anti-rabbit antibody IgG for 20 min at room temperature and then for 30 min with Streptavidin-HRP peroxidase. Diaminobenzidine (DAB)-H_2_O_2_ was used as a substrate for the peroxidase enzyme.

### Statistical analysis

All statistical analyses were conducted using SPSS software (version 17.0, SPSS, Inc., Chicago, IL, USA) and GraphPad Prism software (Version 5.0, GraphPad Prism Software Inc., San Diego, CA). The data are presented as the mean ± standard deviation (SD). Statistical significance between normal and tumor tissues was determined using the non-parametric Mann–Whitney U-test. Statistical analysis between two groups was conducted using Two-tailed unpaired Student's t-test. The relationship between miR-195-5p expression level and clinicopathologic factors was evaluated using Pearson's Chi-square test. Patient survival was evaluated using the Kaplan-Meier method and compared using logrank test. Significant differences were obtained for a p value less than 0.05 (*p < 0.05).
